# INFLAMMATORY BOWEL DISEASES: CHARACTERISTICS, EVOLUTION, AND QUALITY OF LIFE

**DOI:** 10.1590/0102-672020210002e1653

**Published:** 2022-06-17

**Authors:** Caique Moraes MENDONÇA, Isaac José Felippe Correa, Alexander de Sá ROLIM, Laercio ROBLES

**Affiliations:** 1 Faculty of Medicine Santa Marcelina - Sao Paulo - SP - Brazil ;; 2 Santa Marcelina Hospital , Coloproctology Unit- São Paulo - SP - Brazil.

**Keywords:** Hospitalization, Quality of Life, Crohn Disease, Colitis, Ulcerative, Hospitalização, Qualidade de Vida, Doença de Crohn, Colite Ulcerativa

## Abstract

Inflammatory bowel diseases present progressive and potentially debilitating characteristics with an impact on health-related quality of life (QoL) throughout the course of the disease, and this parameter may even be used as a method of evaluating response to treatment.

**AIM:**

The aim of this study was to analyze epidemiological data, medications in use, previous surgeries, and hospitalizations in patients with inflammatory bowel diseases, and to determine the impairment in QoL of these patients.

**METHODS:**

This is a prospective, cross-sectional, observational study in patients with inflammatory bowel disease followed up in a tertiary hospital in São Paulo-SP, Brazil. General and disease-related, evolution, and quality-of-life data were analyzed using a validated quality-of-life questionnaire, namely, Inflammatory Bowel Disease Questionnaire (IBDQ).

**RESULTS:**

Fifty-six individuals were evaluated, with an equal number of patients with Crohn’s disease and ulcerative colitis. A higher prevalence of previous surgeries (p=0.001) and hospitalizations (p=0.003) for clinical-surgical complications was observed in patients with Crohn’s disease. In addition, the impairment of QoL also occurred more significantly in these patients (p=0.022), and there was a greater impact on females in both forms of inflammatory bowel disease (p=0.005).

**CONCLUSIONS:**

Patients with Crohn’s disease are more commonly submitted to surgeries and hospitalizations. Patients affected by both forms of inflammatory bowel disease present impairments in QoL, which are mainly related to intestinal symptoms, and females are more affected than men.

## RESUMO - RACIONAL

As doenças inflamatórias intestinais apresentam características progressivas e potencialmente debilitantes com impacto na qualidade de vida relacionada à saúde durante todo o curso da doença, podendo esse parâmetro inclusive ser utilizado como método de avaliação da resposta ao tratamento.

**OBJETIVO:**

Analisar dados epidemiológicos, medicamentos em uso, cirurgias e internações prévias em pacientes com doenças inflamatórias intestinais e determinar o comprometimento na qualidade de vida desses pacientes.

**MÉTODOS:**

Estudo prospectivo, transversal e observacional em portadores de doença inflamatória intestinal acompanhados em hospital de ensino de São Paulo-SP. Foram analisadas as características gerais e relacionados às doenças, evolução e qualidade de vida utilizando um questionário validado, o *Inflammatory Bowel Disease Questionnaire* (IBDQ).

**RESULTADOS:**

Cinquenta e seis indivíduos foram avaliados, com igual número de pacientes com Doença de Crohn e Retocolite Ulcerativa. Foi observada maior prevalência de cirurgias prévias (p=0,001) e de internações por complicações clínico-cirúrgicas em portadores de doença de Crohn (p=0,003). Além disso, o prejuízo da qualidade de vida também ocorreu de forma mais relevante nesses pacientes (p=0,022) e houve maior impacto no sexo feminino em ambas as formas de doença inflamatória intestinal (p=0,005).

**CONCLUSÃO:**

Os portadores de doença de Crohn são mais comumente submetidos a cirurgias e internações. Os pacientes acometidos por ambas as formas de doença inflamatória intestinal apresentam prejuízos na qualidade de vida, principalmente relacionados aos sintomas intestinais e de forma mais negativa no sexo feminino.

## INTRODUCTION

Inflammatory bowel disease (IBD) refers to a group of diseases [Crohn’s disease (CD) and ulcerative colitis (UC)] that are autoimmune, chronic, and of unknown etiology^25^ ^,^ ^27^. Moreover, it presents progressive and potentially debilitating characteristics^24^.

The study of health-related quality of life (HRQoL) in these patients is relevant, as it can lead to changes in the social, psychological, and social-professional spheres and, during the course of the disease, several factors that interact and combine can cause different impacts on the degree of life satisfaction of these individuals and can even be used as a method to evaluate the treatment^2^ ^,^ ^18^.

Symptoms such as profuse chronic diarrhea, abdominal pain, and hyporexia affect HRQoL.^3^ Therapeutic measures, whether conservative (adverse drug effects) or surgical (resections, definitive ostomies) , result in frequent adverse effects in the long term^14^ ^,^ ^24^, affecting the life of patient mentally, emotionally, physiologically, socially, and physically^4^.

The objective was to cross-sectionally analyze epidemiological data, medications in use, previous surgeries, and hospitalizations in patients with IBDs at the Coloproctology Unit of the Department of General Surgery of Santa Marcelina Hospital and determine the impairment of QoL of these patients and the aspects involved in this impairment.

## METHODS

This is a prospective, cross-sectional, observational study involving patients with IBD (CD and UC) followed at the Coloproctology Unit of the Department of General Surgery of Santa Marcelina Hospital, from March 2019 to February 2020.

Patients over 18 years of age, with physical and mental capacity to participate in the study and agree with the term of consent and participation in the work, were included. Pregnant women and patients with an indication for emergency hospitalization were excluded.

The following items were analyzed:

1. General data: age, sex, race, marital status, education, body mass index (BMI), smoking, and comorbidities
2. Disease-related data: length of disease, previous hospitalization due to clinical and/or surgical complications, and medications in use
3. Application of IBD Life Questionnaire: IBDQ (Intestinal Bowel Disease Questionnaire) ^7^ ^,^ ^8^ ^,^ ^13^ ^,^ ^16^ ^,^ ^18^ ^,^ ^19^ ^,^ ^20^ ^,^ ^25^ ^,^ ^27^, which contains 32 items comprising four domains:
  - **Intestinal symptoms** (10 questions - 01, 05, 09, 13, 17, 20, 22, 24, 26, 29; ranging from 10 to 70 points): frequency of bowel movements; diarrhea; abdominal cramping; discomfort from pain in the belly; problem with eliminating large amounts of gas; feeling of bloating in the belly; rectal bleeding on bowel movements; discomfort from going to the toilet to evacuate and not being able to despite effort and from accidentally evacuating in the pants; feeling of nausea
  - **Systemic symptoms** (five questions - 02, 06, 10, 14, 18; ranging from 5 to 35 points): feeling of tiredness, fatigue, exhaustion; physical tiredness; feeling of malaise; sleep disturbance because of intestinal problem; problem to maintain weight as you would like it to be
  - **Emotional aspects** (12 questions - 03, 07, 11, 15, 19, 12, 23, 25, 27, 30, 31, 32; ranging from 12 to 84 points): frequency of feeling frustrated, impatient, or restless; worried about the possibility of needing an operation for the bowel problem; frequency that one has felt depressed and lacking courage; frequency that one feels worried or anxious; how long one has felt calm and relaxed; embarrassment because of the bowel problem; urge to cry; anger over the bowel problem; how long one has felt angry; lack of understanding from other people; how satisfied, happy, or grateful one feels about their personal life.
  - **Social aspects** (five questions - 04, 08, 12, 16, 28; ranging from 5 to 35 points): inability to go to school or work because of the bowel problem; need to delay or cancel social engagements; difficulty doing sports or having fun as one would like to because of the bowel problem; avoid going to places that do not have toilets nearby; avoid sexual activity because of the bowel problem.

The score of the answers was presented through multiple choice with seven alternatives (Likert scale), with each question ranging from 1 (representing a “worst” aspect) to 7 (representing a “best” aspect), so that the total IBDQ score is between 32 and 224; the lower the score, the greater the impact on QoL^3^ ^,^ ^21^ ^,^ ^26^.

The author provided the informed consent form and then the questionnaire to the participant. If the participant had any doubts, the interviewer repeated the wording of each question to reinforce the understanding of the interviewee. The study was evaluated and approved by the Faculty of Medicine Santa Marcelina’s Research Orientation Committee (COPE-FASM: opinion number P010/2019), by Plataforma Brasil, and the consubstantiated opinion of the ROC (number: 3.574.576).

Statistical analyses were performed using the IBM SPSS Statistics version 20 software for Windows, and Pearson’s correlation analysis was used with a power of 95% and alpha probability fixed at 5%. The scatter diagram was used to verify the relationship between various aspects and total score.

The differences between the total IBDQ of comparative groups of CD and UC were combined using the Student’s t-test. The verification of homogeneity or heterogeneity of variances was carried out by one-tailed F test. For the stratification of data related to sex, the dummy binary variable was created. The dispersion diagram with the curve plot for a trend line and ANOVA was used to indicate which of the symptoms best fit the IBDQ score (Crohn’s alpha coefficient for intestinal, systemic, emotional, social, and total with 95%), and for all analyses, a value of p=0.05 was considered.

## RESULTS

Fifty-six patients with IBD were evaluated, with equal number of patients with CD and UC, and the demographic characteristics are summarized in Table 1.

**Table 1 -:** General data on the prevalence of IBD, CD, and UC regarding marital status, education, gender, color, smoking, comorbidities, duration of disease, BMI, and age of the patients.

Characteristics	IBD (%)	CD (%)	UC (%)
Sample	56 (100%)	28 (50%)	28 (50%)
Marital status			
Single	23 (41.07%)	13 (46.42%)	10 (35.71%)
Married	25 (44.6%)	13 (46.42%)	12 (42.85%)
Divorced	3 (5.35%)	0 (0%)	3 (10.71%)
Widower	5 (8.9%)	2 (7.14%)	3 (10.71%)
Education			
Less or equal to primary education	17 (30.35%)	7 (25%)	10 (35.7%)
Less or equal to secondary education	25 (44.64%)	13 (46.5%)	12 (42.85%)
Less or equal to undergraduate degree	14 (25%)	8 (28.5%)	6 (21.4%)
Gender			
Male	26 (46.4%)	11 (39.3%)	15 (53.6%)
Female	30 (53.6%)	17 (60.7%)	13 (46.4%)
Race			
Caucasian	34 (60.7%)	16 (57.1%)	18 (64.3%)
African-American	7 (12.5%)	2 (7.1%)	5 (17.9%)
Brown	15(26.7%)	10 (35.7%)	5 (17.9%)
BMI (kg/m^2^): Average ± SD	25,7 ± 4,98	24,59 ± 5,03	26,85 ± 4,76
Age (years): Average ± SD	45.93 ± 17.5	41.21 ± 15.85	50.64 ± 18.1
Smoking			
Yes	3 (5.4%)	0 (0%)	3 (10.7%)
No	53 (94.6)	28 (100%)	25 (89.3%)
Comorbities			
No	32 (57.1%)	18 (64.3%)	14 (50%)
Yes	24 (42.9%)	10 (35.7%)	14 (50%)

**Figure 1 -:**
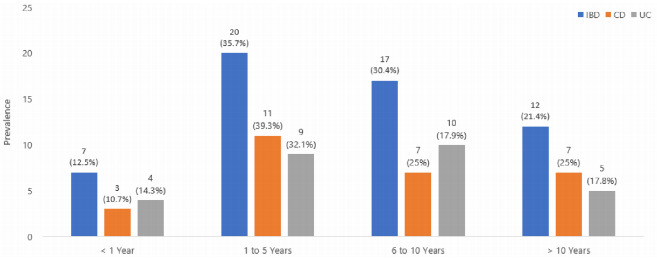
Prevalence of IBD and CD and UC forms in relation to the time of disease.

Figure 1 shows the prevalence of IBD and of the CD and UC forms in relation to the time of disease, where it can be seen that in most of the study patients the prevalence of disease was found between 1 and 10 years.

Regarding previous surgeries, we observed that 23 patients with IBD (41.1%) had undergone some surgical procedures, mostly intestinal. Of the patients with CD, 67.9% had already undergone surgery, whereas among the patients with UC, only 14.2% had undergone surgery (p=0.001) (Table 1). Notably, 27 (48.2%) patients with IBD, 19 (67.9%) patients with CD, and 8 (28.6%) patients with UC (p=0.003) required hospitalization for clinical-surgical complications(Table 2).

**Table 2 -:** Prevalence data of CD and UC regarding previous surgeries and hospitalization for clinical-surgical complication.

Characteristic	IBD (%)	CD (%)	UC (%)
Previous surgeries			
No	33 (58.9%)	9 (32.1%)	24 (85.7%)
Colorectal	15 (26.8%)	13 (46.1%)	2 (7.1%)
Colorectal and orificial	1 (1.8%)	1 (3.6%)	0 (0%)
Orificial	7 (12.5%)	5 (17.9%)	2 (7.1%)
Hospitalization for clinical-surgical complication			
Yes	27 (48.2%)	19 (67.9%)	8 (28.6%)
No	29 (51.8%)	9 (32.1%)	20 (71.4%)

Table 3 stratifies the surgeries according to the length of disease, where it is possible to verify that most surgical procedures occurred in both diseases between 5 and 10 years of diagnosis, notably in patients with CD (p=0.003).

**Table 3 -:** Prevalence of previous surgeries in relation to the length of disease.

Previous surgeries and duration of disease				
Length of disease	Previous surgeries			
	Number (%)	Colorectal (%)	Colorectal orificial (%)	Orificial (%)
IBD				
Up to 4 years	8 (24.2%)	6 (40%)	0 (0%)	4 (57.1%)
5-10 years	18 (54.5%)	5 (33.3%)	0 (0%)	3 (42.9%)
>10 years	7 (21.2%)	4 (26.7%)	1 (100%)	0 (0%)
Total	33 (100%)	15 (100.0%)	1 (100%)	7 (100%)
CD				
Up to 4 years	2 (22.2%)	5 (38.5%)	0 (0%)	3 (60%)
5-10 years	5 (55.6%)	4 (30.8%)	0 (0%)	2 (40%)
>10 years	2 (22.2%)	4 (30.8%)	1 (100%)	0 (0%)
Total	9 (100%)	13 (100%)	1 (100%)	5 (100%)
UC				
Up to 4 years	2 (22.2%)	5 (38.5%)	0 (0%)	3 (60%)
5-10 years	5 (55.6%)	4 (30.8%)	0 (0%)	2 (40%)
>10 years	2 (22.2%)	4 (30.8%)	1 (100%)	0 (0%)
Total	9 (100%)	13 (100%)	1 (100%)	5 (100%)

Regarding pharmacological therapy, the following drugs are used: aminosalicylates in 27 (48.2%), 5 (17.9%), and 22 (78.6%) patients with IBD, corticosteroids in 9 (16.1%), 5 (16.9%), and 4 (14.3%) patients with CD, immunosuppressants in 13 (23.2%), 11 (39.3%), and 2 (7.1%) patients with UC, and immunobiologicals in 25 (44.6%), 22 (78.6%), and 3 (10.7%) patients.

Table 4 presents the results of analysis of the IBDQ domains in IBD, CD, and UC using Student’s t-test, where one can observe a higher index of global, bowel, and systemic symptoms in UC, and thus a lower impact on QoL of patients (p=0.022).

**Table 4 -:** Domains of the global IBDQ in IBD, CD, and UC

IBDQ Score	IBD (n=56)	CD (n=28)	UC (n=28)	p-value
Intestinal symptoms	52.39±14.05	49.21±16.69	55.57±10.13	0.045
Systemic symptoms	23.84±8.54	21.11±9.50	26.57±6.55	0.007
Emotional aspects	56.00±19.54	52.82±21.33	60.85±17.00	0.060
Social aspects	24,55±9,05	22.00±9.77	27.10±7.60	0.016
Total IBDQ	157.63±46.67	145.14±52.77	170.1±36.47	0.022

Table 5 stratifies the values of IBDQ questionnaire in IBD, CD, and UC and correlates them with gender using Student’s t-test, where we observed a higher score in males. Table 6 stratifies the domains in CD and UC and shows comparison between genders.

**Table 5 -:** IBDQ index score in IBD, CD, and UC comparing genders

Score	Average + SD		
Total IBDQ	IBD		p-value
	178.54±37.72	139.51±46.61	0.0005
	CD		p-value
	175.82±44.13	125.29±49.15	0.004
	UC		p-value
	180.53±33.76	158.08±37.02	0.05

**Table 6 -:** Stratification of IBDQ domains in CD and UC and comparison between genders.

Domains	CD			UC		
	Males	Females	p-value	Males	Females	p-value
Intestinal	59.45±13.67	42.59±15.32	0.002	57.47±9.90	53.38±10.34	0.149
Systemic	26.82±7.62	17.41±8.89	0.0032	29.00±5.27	23.77±6.95	0.018
Emotional	63.64±17.16	45.82±21.24	0.011	65.60±13.87	55.38±19.14	0.0625
Social	25,91 ± 8,65	19.47±9.85	0.04	28.47±8.11	25.54±6.96	0.156

**Figure 2 -:**
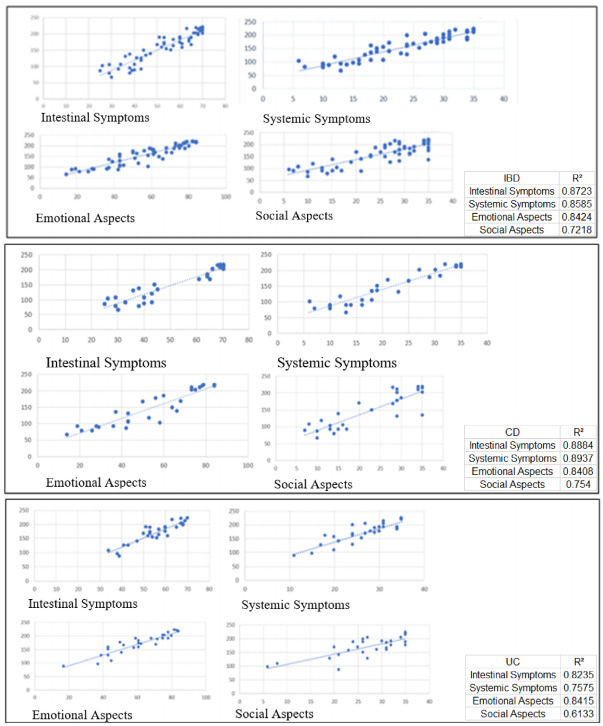
Scatter diagram showing the relationship between various aspects and total score.

The scatter diagram (Figure 2) was used to verify the relationship between the various aspects and the total score where a higher R² value corresponds to a more accurate adjustment. It can be seen that for the population with IBD, the characteristic that best explained the total IBDQ score was that of intestinal symptoms and the one that explained the least was that of social aspects.

## DISCUSSION

This was a prospective study assessed in a sample of patients with IBD who were followed up in a specialized tertiary hospital in São Paulo, Brazil. We analyzed a number of patients consistent with some studies in the literature on the subject and also the distribution of the two forms of the disease^16^ ^,^ ^17^.

Regarding demographic data, we observed an overall mean age similar to the literature^1^ ^,^ ^16^ ^,^ ^19^ ^,^ ^20^; likewise, the mean duration of disease in this study was similar to the studies surveyed^13^ ^,^ ^15^ ^,^ ^17^ for a period of more than 5 years.

According to the literature^17^ ^,^ ^18^ ^,^ ^20^, we observed a higher percentage of surgeries among patients with CD (p=0.001); however, with more frequent abdominal surgeries reported in different studies, orificial procedures were notably observed^22^ ^,^ ^28^. Moreover, hospitalizations related to morbidity were also numerous in these patients (p=0.003), which was consistent with the literature^5^ ^,^ ^7^ ^,^ ^10^ ^,^ ^17^ ^,^ ^18^ ^,^ ^20^.

Regarding drug therapy, it was noted that among patients with UC, mainly aminosalicylates were used, while biological drugs were mainly used in treating patients with Crohn’s disease, matching the literature surveys^5^ ^,^ ^9^ ^,^ ^11^ ^,^ ^16^.

Table 7 compares previous studies with this study and we can observe an acceptable number of patients for the analysis of the impact of IBD on QoL. In addition, it can be seen that the gut and systemic symptom scores of this study are close to those reported by Han et al.^13^ and Pallis et al.^17^

**Table 7 -:** Comparison between IBDQ scores among studies

Domains	Data in this study (n=56)	Han et al.^13^ (n=30)	Boer et al.^7^ (n=271)	Pallis et al.^17^ (n=135)
Intestinal	52.39±14.05	54.9±10.4	37.3±7.7	58.9±10.7
Systemic	23.84±8.54	25.3±5.9	17.0±4.4	27.7±6.9
Social	56.0±19.54	29.4±8.1	20.0±4.7	29.1±7.5
Emotional	24.55±9.05	64.1±13.7	44.9±9.1	62.4±15.6
Total	157.63±46.67	173.7±33.1	119.1±22.0	178.1±36.9

Although the scores for the social and emotional aspects differ from those found in these publications, it is noticeable that the total IBDQ score of this study falls between the ranges^13^ ^,^ ^17^. Van der Eijk et al.^23^ showed that psychological stress, including anxiety, depression, and stressful life events, has a negative impact on the QoL of patients with IBD.

In this study, female patients with IBD, CD, and UC had lower IBDQ scores when compared to males (p=0.0005, 0.004, and 0.05, respectively), inferring that they have a greater impairment in QoL, which is consistent with the study by Magalhães et al.^16^ who showed that women with CD presented significantly lower IBDQ score than men (p=0.023). However, the results of same study differ from ours in patients with UC, as we also observed a greater impact on QoL in females (p=0.05), whereas in the cited work, we did not observe a significant impact on QoL (p=0.061).

It was also observed in the present study that the total IBDQ score is more affected in patients with Crohn’s disease (p=0.022), which is consistent with the literature^6^ ^,^ ^12^; however, it differs from the studies of some authors which do not show statistical difference^8^ ^,^ ^16^ ^,^ ^20^.

As a limitation of the study, one can think of the relatively small number of patients; however, a prospective collection performed by a single researcher in a university health center is another limitation. Moreover, this study emphasizes the necessary appreciation of the QoL in patients with IBD, looking for a better assistance to these patients with chronic disease.

## CONCLUSIONS

Patients with Crohn’s disease are more commonly submitted to surgeries and hospitalizations with a greater impairment of QoL - notably in females - among patients with IBD.
